# Sequential detection of alphafetoprotein-bearing cells in blood stem cell fraction of germ cell tumour patients

**DOI:** 10.1054/bjoc.2001.2050

**Published:** 2001-10

**Authors:** T Kasahara, N Hara, V Bilim, Y Tomita, K Saito, K Obara, K Takahashi

**Affiliations:** Division of Molecular Oncology, Department of Signal Transduction Research, Division of Urology, Department of Regenerative and Transplant Medicine, Niigata University Graduate School of Medical and Dental Sciences, Asahimachi 1-757, Niigata, 951-8510, Japan

**Keywords:** germ cell tumour, alphafetoprotein, peripheral blood stem cells, reverse transcription polymerase chain reaction

## Abstract

High-dose chemotherapy with peripheral blood stem cell (PBSC) transplantation in advanced germ cell tumour (GCT) patients is widely applied. The aims of this study were: (1) To examine the presence of alphafetoprotein (AFP) bearing tumour cells in PBSC harvests from advanced GCT patients obtained after multiple cycles of induction chemotherapy. (2) To determine whether induction chemotherapy contributed to in vivo purging of the tumour. We evaluated cryopreserved PBSC samples from 5 patients with advanced stage II/III AFP producing GCT. PBSC were separated after the first, second and third cycles of induction chemotherapy. Those samples were analysed using the nested reverse transcription polymerase chain reaction (RT-PCR) method to detect AFP mRNA. Although, in all patients, AFP mRNA was detected in PBSC samples after the first or second cycle of induction chemotherapy, but was not detected in 3 of 4 samples after the third cycle of chemotherapy. Although it is not clear whether tumour cells contaminating PBSC fraction contribute to disease relapse, PBSC harvested after at least 3 cycles of induction chemotherapy might be recommended to avoid such a possibility. © 2001 Cancer Research Campaignhttp://www.bjcancer.com


British Journal of Cancer (2001) 85(8), 1119–1123
© 2001 Cancer Research Campaign
doi: 10.1054/ bjoc.2001.2050, available online at http://www.idealibrary.com on IDEAL

http://www.bjcancer.com

### Sequential detection of alphafetoprotein-bearing cells in blood stem cell fraction of germ cell tumour patients

**T Kasahara[1,2], N Hara[1], V Bilim[1], Y Tomita[1], K Saito[2], K Obara[2] and K Takahashi[2]**

[1]Division of Molecular Oncology, Department of Signal Transduction Research and [2]Division of Urology, Department of Regenerative and Transplant Medicine, Niigata University Graduate School of Medical and Dental Sciences, Asahimachi 1-757, Niigata 951-8510, Japan

**Summary** High-dose chemotherapy with peripheral blood stem cell (PBSC) transplantation in advanced germ cell tumour (GCT) patients is widely applied. The aims of this study were: (1) To examine the presence of alphafetoprotein (AFP) bearing tumour cells in PBSC harvests from advanced GCT patients obtained after multiple cycles of induction chemotherapy. (2) To determine whether induction chemotherapy contributed to in vivo purging of the tumour. We evaluated cryopreserved PBSC samples from 5 patients with advanced stage II/III AFP producing GCT. PBSC were separated after the first, second and third cycles of induction chemotherapy. Those samples were analysed using the nested reverse transcription polymerase chain reaction (RT-PCR) method to detect AFP mRNA. Although, in all patients, AFP mRNA was detected in PBSC samples after the first or second cycle of induction chemotherapy, but was not detected in 3 of 4 samples after the third cycle of chemotherapy. Although it is not clear whether tumour cells contaminating PBSC fraction contribute to disease relapse, PBSC harvested after at least 3 cycles of induction chemotherapy might be recommended to avoid such a possibility. © 2001 Cancer Research Campaign http://www.bjcancer.com

**Keywords**: germ cell tumour; alphafetoprotein; peripheral blood stem cells; reverse transcription polymerase chain reaction

Autologous transplantation of peripheral blood stem cells (PBSC), harvested following priming chemotherapy and granulocyte colony-stimulating factor (G-CSF), can endure myeloablative doses of anti-cancer drugs (Elias et al, 1995; Fields et al, 1995). High-dose chemotherapy (HDC) combined with autologous bone marrow transplantation (BMT) or PBSC transplantation (PBSCT) has considerable efficacy for the treatment of patients with testicular germ cell tumours (GCT) who do not achieve a disease-free state after conventional-dose chemotherapy (Nichols et al, 1992; Beyer et al, 1997; Broun et al, 1997). PBSCT has been increasingly used in place of BMT. The advantages of this method over BMT are (1) no need to perform collection under general anaesthesia, (2) the possibility of harvesting progenitor cells in patient with bone marrow fibrosis resulting from pelvic irradiation and (3) more rapid haematopoietic recovery (Armitage, 1994).

However, one concern is that inadvertent reinfusion of tumour cells contaminating the preparations of PBSC may contribute to disease relapse. Reinfusion of malignant cells in autologous BMT has been shown to contribute to disease recurrence in some haematological tumours (Brenner et al, 1993; Rill et al, 1994). Although PBSCT is believed to have a lower risk of contamination by tumour cells (range 4–20%) than BMT according to some previous studies using histological or immunocytochemical techniques (Fields et al, 1996; Passos-Coelho et al, 1996; Schulze et al, 1997), recent studies demonstrated a higher frequency than expected, adopting more sensitive reverse transcription polymerase chain reaction (RT-PCR) based methods for detecting mRNA of tumour cell-derived products (Mattano et al, 1992; Craig et al, 1994; Vannucchi et al, 1998).

*Received 6 February 2001*
*Revised 3 July 2001*
*Accepted 13 July 2001*

*Correspondence to:* Y Tomita

In the present study, using nested RT-PCR for detecting alphafetoprotein (AFP) mRNA, we obtained evidence of tumour cell contamination in PBSC fractions from advanced GCT patients. Furthermore, we also found that the amount of AFP mRNA decreased with repeated cycles of combined chemotherapy.

### MATERIALS AND METHODS

### Patient samples

We evaluated, retrospectively, PBSC frozen samples from 5 patients with advanced testicular GCT and elevated serum AFP (range 26–44 years old, mean age 32). The clinical stages and pathological diagnoses are shown in Table 1. All patients had visceral involvement and/or lymph node (LN) metastasis. They had undergone orchiectomy, and received 3 cycles of a conventional chemotherapy regimen, EP or BEP (VP-16 100 mg $m^{-2}$, cisplatin 20 mg $m^{-2}$ and bleomycin 30 U) (Table 2). Mobilization of haematopoietic stem cells in the peripheral blood was performed by daily injections of recombinant G-CSF (75 μg $m^{-2}$ filgrastim/$day^{-1}$, s.c. or 100 μg $m^{-2}$ lenograstim $day^{-1}$, s.c.) until the number of leukocytes reached 10 000 $\mu l^{-1}$. Mononuclear cells (MNC) were collected using an AS104 separator, and 7.2 litres of blood was processed per leukopheresis. Each MNC fraction was centrifuged, resuspended in albumin, DMSO and starch mixture, and stored at –80˚C until use. Two or 3 consecutive leukophereses per cycle were performed to harvest a total of more than $2 \times 10^6$ CD34 + cells per kg of body weight.

**Table 1** Characteristics, at diagnosis, of patients with germ cell tumours

| Patient no. | Age (years) | Testis cancer stage (UICC) | Histological analysis | Site of metastasis |
|---|---|---|---|---|
| 1 | 34 | IIIC | EC>T>S | RP, Liver |
| 2 | 27 | IIIC | EC+T+CC | RP, Liver |
| 3 | 44 | IIB | S>EC | RP |
| 4 | 26 | IIIA | EC+YST+CC+T | RP, Supraclavicular LN |
| 5 | 31 | IIIC | EC | RP, Axillary LN, Maxillary sinus |

EC = embryonal carcinoma; T = teratoma; S = seminoma; CC = choriocarcinoma; YST = yolk sac tumour; RP = retroperitoneal lymph node; LN = lymph node.

**Table 2** Prior treatment and serum AFP level

| Patient no. | Induction chemotherapy | Response | Serum AFP (normal value < 10 ng ml$^{-1}$) | | | |
|---|---|---|---|---|---|---|
| | | | Diagnosis | Harvest 1 | Harvest 2 | Harvest 3 |
| 1 | EPX3 | PR | 16 763 | 10 131 | 820 | ND |
| 2 | EPX3 | PR | 1851 | 1156 | 53 | 12 |
| 3 | EPX3 | PR | 29 | 11 | 5 | 5 |
| 4 | EPX3 | NC | 110 | 29 | 31 | 22 |
| 5 | BEPX3 | PR | X | 10 | ND | <1 |

Patients described in Table 1. EP: VP-16, cisplatin; BEP: bleomycin, VP-16, cisplatin; CR = complete remission; PR = partial remission; NC = no change; Harvest 1–3: harvest during the first, second and third cycle of chemotherapy, respectively; X: not measured; ND: PBSC apheresis during that cycle was not performed.

**Table 3** Nested RT-PCR results and clinical course after PBSCT

| Patient no. | 1st chem. | 2nd chem. | 3rd chem. | Transplanted PBSC | Status |
|---|---|---|---|---|---|
| 1 | (+) | (+) | NA | Harvest 2 (2 of 2 subsets) | Dead[a] |
| 2 | (+) | NA | (+) | Harvest 1 (2 of 2 subsets) | NED |
| 3 | NA | (+) | (–) | Harvest 3 (2 of 3 subsets) | NED |
| 4 | NA | (+) | (–) | Harvest 3 (1 of 2 subsets) | NED |
| 5 | (+) | NA | (–) | ND | Dead[b] |

(+): AFP mRNA positive; (–): AFP mRNA negative; NA: sample not available; Harvest: PBSCs described in Table 2; ND: not done; [a]: died 3 days after PBSCT due to infection; [b]: died due to carcinomatous meningitis.

### Autologous stem cell transplantation

After conventional chemotherapy, 4 of 5 patients were treated with HDC, which consisted of VP-16 (320 mg m$^{-2}$), ifosfamide (2 g m$^{-2}$) and carboplatin (320 mg m$^{-2}$) on days -6 to -2 (patient 5 died before HDC due to carcinomatous meningitis). At the time of transplant, one or two subsets of harvested cells (total number of CD34+ cells was $2 \times 10^6$ per kg of body weight) were reinfused to each patient (Table 3).

### RNA isolation from PBSC harvests

RNA was isolated from 1 ml of processed fraction containing approximately $1 \times 10^7$ MNC, which was aliquoted at PBSC collection. Cells were thawed in a 37˚C water bath, and wahsed once in sterile phosphate-buffered saline (PBS). Cells were processed using the SV total RNA isolation system (Promega, Madison, WI) according to the manufacturer's instructions, and resuspended in 100 µl of diethyl pyrocarbonate-treated (DEPC) water.

### Cell line

The Hep 3B human hepatocellular carcinoma cell line, which secretes AFP, was used as a control (Knowles et al, 1980; Jiang et al, 1997). The cell line was kindly provided by Dr Toshio Kudo (Institute of Development, Aging and Cancer, Tohoku University, Sendai, Japan). Cells were cultured in RPMI-1640 medium supplemented with 10% fetal calf serum in 5% $CO_2$/ 95% air at 37˚C. To check the sensitivity of the assay, $1 \times 10^7$ peripheral MNC from a healthy volunteer were mixed with serial dilutions of Hep 3B cells ($1 \times 10^3$–$10^0$).

### Nested RT-PCR amplification of AFP mRNA

cDNA synthesis and RT-PCR were performed as described previously (Bilim et al, 1998). Briefly, aliquots of 8.2 µl RNA were used and synthesis of cDNA was performed in a 20-µl reaction using a first-strand cDNA synthesis kit (Boehringer Mannheim, Indianapolis, IN). The mixture was incubated at 25˚C for 10

*© 2001 Cancer Research Campaign*

minutes and then at 42˚C for 60 minutes. The reaction was stopped by enzyme inactivation at 95˚C for 5 minutes. Before analysis, amplification of the constitutively expressed β-actin gene was performed to ensure appropriate first-strand cDNA synthesis.

The nested PCR was conducted by adding 1 μl of cDNA to 50 μl of reaction buffer (10 mM Tris-HCl, pH 8.3, 50 mM KCl, 1.5 mM $MgCl_2$ and 200 mg $ml^{-1}$ gelatin), with 200 μM of each dNTP, 400 nM of each outer primer and 2.5 units of AmpliTaq Gold™ (Perkin Elmer, Branchburg, NJ). 40 cycles of 1 min denaturation at 94˚C, 1 min annealing at 52˚C and 1 min extension step at 72˚C were performed in a thermal cycler (Perkin Elmer). A sample of 1 μl of the first amplification product was subjected to the nested amplification using inner primer pairs with the same reagents and protocol. The amplified products were analysed by electrophoresis on a 3% agarose gel. Two sets of primers specific for the AFP cDNA sequence were designed (Morinaga et al, 1983). The outer pair of primers were sense: 5′-ACTGAATCCA-GAACACTGCATAG-3′ (nucleotides 90–112, in exon 1) and antisense: 5′-TGCAGTCAATGCATCTTTCACCA-3′ (nucleotides 263–241, in exon 3). The inner pair of primers were sense: 5’-TGGAATAGCTTCCATATTGGATTC-3′ (nucleotides 122–145, over exon 1 and exon 2, antisense: 5′-AAGTGGCTTCTTGAA-CAAACTGG-3′ (nucleotides 222–200, in exon 3) (Komeda et al, 1995). Since the inner sense primer was designed to bridge the intron, the competition of contaminating genomic DNA could be eliminated. The identity of the amplified products was confirmed by direct sequencing (data not shown). Each PCR reaction was performed in triplicate to confirm the accuracy of the findings.

### RESULTS

### Specificity and sensitivity of the nested RT-PCR assay for AFP

Using cDNA from Hep 3B cells, which secrete AFP, specific bands (101 bp) were detected (Figure 1). Peripheral MNC obtained from healthy volunteers (approximately $1 \times 10^7$ cells) were used as the negative control. We also examined PBSC fractions from 3 other patients whose primary testicular tumours did not contain AFP-producing cells (two of them were diagnosed as seminoma and one was choriocarcinoma). No detectable mRNA was found in the harvests obtained after any cycle of conventional chemotherapy (data not shown).

The sensitivity of the nested PCR was determined by simulating a contamination model with a different number of Hep 3B cells into MNC obtained from a healthy volunteer. As few as 10 Hep 3B cells were detected among $1 \times 10^7$ MNC (Figure 1).

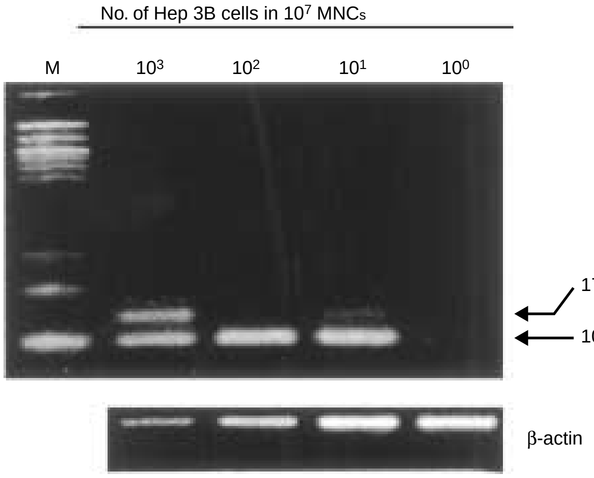

**Figure 1** Sensitivity of nested PCR for detection of AFP mRNA. Peripheral mononuclear cells ($1 \times 10^7$) were mixed with $10^3$, $10^2$, $10^1$ and $10^0$ Hep 3B cells. RNA was isolated from each mixture, and cDNA was prepared using random primers. Nested PCR using AFP primers was performed. The 176 and 101 base pair fragments were amplified from outer and inner primer pairs, respectively (upper). In all samples, the presence of adequately amplified mRNA had been assessed by RT-PCR of the β-actin gene (lower). Lane M: 100 base pairs DNA marker (New England Biolabs, Massachusetts)

### Nested PCR analysis of PBSC fractions from AFP producing GCT patients

The patient characteristics are summarized in Table 1. In the primary tumours, the presence of embryonal carcinoma (EC) and/or yolk sac tumour (YST), either of which may produce AFP, was confirmed histopathologically. In all patients, AFP mRNA was detected in PBSC fractions harvested after the first and second cycle of conventional chemotherapy. In contrast, in 3 of 4 samples obtained after the third cycle, AFP mRNA was not detected (Figure 2, patients 3–5).

### Comparison between AFP mRNA detection and serum AFP levels

PBSC apheresis during elevated serum AFP (over 10 ng $ml^{-1}$) resulted in detection of AFP mRNA-bearing cells in PBSC fractions in all cases except one. Patient 4 was the only exception. His serum AFP remained high even after HDC, but AFP mRNA in PBSC fraction after the third cycle of induction chemotherapy was not detected. On CT he had cystic residual tumours. Surgical resection of the residual tumours revealed a cystic mass without viable cancer cells, which contained fluid with a high concentration of AFP, leading to elevated serum AFP. As for patient 3, serum AFP decreased to within the normal range by the time of the second cycle of chemotherapy, but AFP mRNA was detected in the PBSC fraction obtained after that cycle.

### Clinical course after HDC with PBSCT

Patient 5, who had multiple LNs and maxillary sinus metastasis, did not receive HDC because he had achieved clinical complete

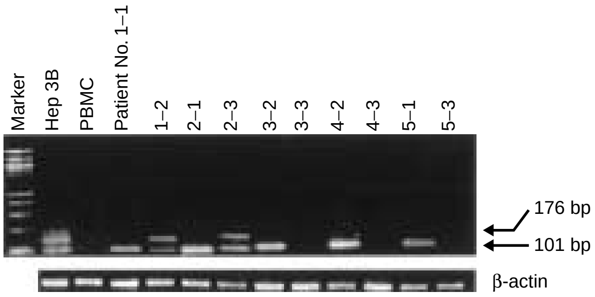

**Figure 2** Detection of AFP mRNA in PBSC fractions from advanced GCT patients and peripheral blood mononuclear cells (PBMC) from a healthy volunteer, using nested RT-PCR (upper). The amplification of the β-actin gene by RT-PCR (lower). Marker: 100 base pairs DNA marker (New England Biolabs). First figure corresponds to a patient; second corresponds to a chemotherapy cycle number

© 2001 Cancer Research Campaign remission by administration of 3 cycles of chemotherapy. However, he died due to carcinomatous meningitis 5 months after the end of chemotherapy without evidence of involvement of other organs. The remaining 4 patients underwent HDC with PBSCT following induction chemotherapy because of partial remission or no clinical response (details of PBSC infusion are shown in Table 3). Patient 1 died during the myelosuppression phase (3 days after PBSCT) due to infection. Two patients (patients 3–4) received resection of residual tumours after HDC. Those tumours did not contain cancer cells histopathologically. Those patients remained alive without disease (mean follow up 36 months). Retrospectively, however, patient 2 was transplanted with AFP mRNA positive fraction (Table 3) although he was alive without evidence of recurrence (more than 5 years).

### DISCUSSION

Although RT-PCR can detect tumour cells with higher sensitivity than the histological or immunocytochemical methods which have been commonly used (Mapara et al, 1997; Vannucchi et al, 1998), low specificity remains a problem (Ghossein et al, 1999). In the present study, to exclude false positive results, MNC from healthy volunteers and PBSC fractions from AFP non-producing GCT patients were examined by nested RT-PCR, but no AFP signals were detected. Moreover, direct sequence of the amplified products of a positive control revealed accurate AFP cDNA.

The sensitivity of this assay was a single tumour cell per $1 \times 10^6$ MNC. We were able to detect circulating AFP-bearing cells even in the fraction where the serum AFP level decreased to within the normal range during chemotherapy. Although the sensitivity of AFP RT-PCR could be further improved (Ghossein et al, 1999), at the present state, it might be a useful modality to predict metastatic disease in germ cell tumours. Besides, detection of AFP mRNA-bearing cells in PBSC fraction may reveal the presence of refractory residual disease after induction chemotherapy.

In all samples harvested after the first and second cycle, AFP mRNA was detected. It was shown that mRNA of β human chorionic gonadotropin (β-HCG), another germ cell tumour marker, was also detected in the PBSC fractions of GCT patients (Fan et al, 1998). Moreover, in the 3 of 4 samples after the third cycle, AFP mRNA was not detected. Our results are in agreement with the clearance of circulating tumour cells in blood samples during chemotherapy in neuroblastoma patients (Moss et al, 1990). These findings imply a possibility of reducing minimal residual tumour cell load (i.e. in vivo purging) by multiple cycles of conventional chemotherapy. Thus, the use of PBSCs after 3 cycles of conventional chemotherapy might reduce the possibility of recurrence due to tumour cell contamination in the fraction, although relapse may take place due to the tumour cells remaining inside the body even after chemotherapy. This is supported by the findings of Vredenburgh et al (1997), who reported that the higher the number of tumour cells identified in the bone marrow harvest, the shorter the disease-free and overall survival.

A variety of techniques have been proposed to purge tumour cells ex vivo (Gribben et al, 1991; Vogel et al, 1996; Spyridonidis et al, 1998). Although prospective randomized trials remain to be investigated to determine whether a positive selection of haematopoietic progenitor cells and/or depletion of tumour cells contaminating PBSC fraction affects clinical outcome, the combination of in vivo and ex vivo purging might become an attractive strategy to improve patient survival.

However, one patient who received PBSCT with AFP mRNA positive cells remained free from recurrence after more than 5 years follow-up. Cooper et al (1998) demonstrated that tumour cells contaminated in PBSC fractions from advanced breast cancer patients were capable of clonogenic growth in vitro, but the authors were unable to find a significant correlation of occult tumour reinfusion with time to progression or overall survival. Tumour cells contaminated in PBSC fractions may not have the capacity to grow in vivo, possibly due to killing by host immune cells (Kennedy et al, 1994). Thus, the presence or absence of AFP mRNA-bearing cells may not necessarily determine recurrence of a tumour after HDC and PBSCT.

In summary, we showed the presence of AFP mRNA-bearing cells in PBSC fractions from GCT patients. The nested RT-PCR method may be useful for detecting circulating tumour cells with high sensitivity. Furthermore, in the administration of HDC with PBSCT, it may assist the determination of the best timing of harvest. Although larger patient groups and different purging trials are required, the present findings suggest that harvesting PBSC after multiple cycles of induction chemotherapy in advanced GCT patients might be recommended in terms of a reduced risk of tumour cell contamination.

### REFERENCES

Armitage JO (1994) Bone marrow transplantation. *N Engl J Med* **330**: 827–837

Beyer J, Kingreen D, Krause M, Schleicher J, Schwaner I, Schwella N, Huhn D and Siegert W (1997) Long-term survival of patients with recurrent or refractory germ cell tumors after high dose chemotherapy. *Cancer* **79**: 161–168

Bilim V, Kawasaki T, Takahashi K and Tomita Y (1998) Absence of frameshift mutations in the bax gene in renal cell cancer (RCC) and transitional cell cancer (TCC). *Anticancer Res* **18**: 1655–1660

Brenner MK, Rill DR, Moen RC, Krance RA, Mirro J, Jr., Anderson WF and Ihle JN (1993) Gene-marking to trace origin of relapse after autologous bone-marrow transplantation. *Lancet* **341**: 85–86

Broun ER, Nichols CR, Gize G, Cornetta K, Hromas RA, Schacht B and Einhorn LH (1997) Tandem high dose chemotherapy with autologous bone marrow transplantation for initial relapse of testicular germ cell cancer. *Cancer* **79**: 1605–1610

Cooper BW, Moss TJ, Ross AA, Ybanez J and Lazarus HM (1998) Occult tumor contamination of hematopoietic stem-cell products does not affect clinical outcome of autologous transplantation in patients with metastatic breast cancer. *J Clin Oncol* **16**: 3509–3517

Craig JI, Langlands K, Parker AC and Anthony RS (1994) Molecular detection of tumor contamination in peripheral blood stem cell harvests. *Exp Hematol* **22**: 898–902

Elias AD, Ayash LJ, Wheeler C, Schwartz G, Tepler I, Gonin R, McCauley M, Mazanet R, Schinipper L and Frei E (1995) Phase I study of high dose ifosfamide, carboplatin and etoposide with autologous hematopoietic stem cell support. *Bone Marrow Transplant* **15**: 373–379

Fan Y, Einhorn L, Saxman S, Katz B, Abonour R and Cornetta K (1998) Detection of germ cell tumor cells in apheresis products using polymerase chain reaction. *Clin Cancer Res* **4**: 93–98

Fields KK, Elfenbein GJ, Lazarus HM, Cooper BW, Perkins JB, Creger RJ, Ballester OF, Hiemenz JH, Janssen WE and Zorsky PE (1995) Maximum tolerated doses of ifosfamide, carboplatin, and etoposide given over 6 days followed by autologous stem-cell rescue: toxicity profile. *J Clin Oncol* **13**: 323–332

Fields KK, Elfenbein GJ, Trudeau WL, Perkins JB, Janssen WE and Moscinski LC (1996) Clinical significance of bone marrow metastases as detected using the polymerase chain reaction in patients with breast cancer undergoing high-dose chemotherapy and autologous bone marrow transplantation. *J Clin Oncol* **14**: 1868–1876

Ghossein RA, Bhattacharya S and Rosai J (1999) Molecular detection of micrometastases and circulating tumor cells in solid tumors. *Clin Cancer Res* **5**: 1950–1960

Gribben JG, Freedman AS, Neuberg D, Roy DC, Blake KW, Woo SD, Grossbard ML, Rabinowe SN, Coral F and Freeman GJ (1991) Immunologic purging of

*© 2001 Cancer Research Campaign*

marrow assessed by PCR before autologous bone marrow transplantation for B-cell lymphoma. *N Eng J Med* **325**: 1525–1533

Jiang SY, Shyu RY, Huang MF, Tang HS, Young TH, Roffler SR, Chiou YS and Yeh MY (1997) Detection of alphafetoprotein-expressing cells in the blood of patients with hepatoma and hepatitis. *Br J Cancer* **75**: 928–933

Kennedy MJ, Vogelsang GB, Jones RJ, Farmer ER, Hess AD, Altomonte V, Huelskamp AM and Davidson NE (1994) Phase I trial of interferon gamma to potentiate cyclosporine-induced graft-versus host disease in women undergoing autologous bone marrow transplantation for breast cancer. *J Clin Oncol* **12**: 249–257

Knowles BB, Howe CC and Aden DP (1980) Human hepatocellular carcinoma cell lines secrete the major plasma proteins and hepatitis B surface antigen. *Science* **209**: 497–499

Komeda T, Fukukda Y, Sando T, Kita R, Furukawa M, Nishida N, Amenomori M and Nakao K (1995) Sensitive detection of circulating hepatocellular carcinoma cells in peripheral venous blood. *Cancer* **75**: 2214–2219

Mapara MY, Korner IJ, Hildebrandt M, Bargou R, Krahl D, Reichardt P and Dorken B (1997) Monitoring of tumor cell purging after highly efficient immunomagnetic selection of CD34 cells from leukapheresis products in breast cancer patients: comparison of immunocytochemical tumor cell staining and reverse transcriptase-polymerase chain reaction. *Blood* **89**: 337–344

Mattano LA, Jr., Moss TJ and Emerson SG (1992) Sensitive detection of rare circulating neuroblastoma cells by the reverse transcriptase-polymerase chain reaction. *Cancer Res* **52**: 4701–4705

Morinaga T, Sakai M, Wegmann TG and Tamaoki T (1983) Primary structures of human alpha-fetoprotein and its mRNA. *Proc Natl Acad Sci USA* **80**: 4604–4608

Moss TJ, Sanders DG, Lasky LC and Bostrom B (1990) Contamination of peripheral blood stem cell harvests by circulating neuroblastoma cells. *Blood* **76**: 1879–1883

Nichols CR, Anderson J, Lazarus HM, Fisher H, Greer J, Stadtmauer EA, Loehrer PJ and Trump DL (1992) High-dose carboplatin and etoposide with autologous bone marrow transplantation in refractory germ cell cancer: an Eastern Cooperative Oncology Group protocol. *J Clin Oncol* **10**: 558–563

Passos-Coelho JL, Ross AA, Kahn DJ, Moss TJ, Davis JM, Huelskamp AM, Noga SJ, Davidson NE and Kennedy MJ (1996) Similar breast cancer cell contamination of single-day peripheral-blood progenitor-cell collections obtained after priming with hematopoietic growth factor alone or after cyclophosphamide followed by growth factor. *J Clin Oncol* **14**: 2569–2575

Rill DR, Santana VM, Roberts WM, Nilson T, Bowman LC, Krance RA, Heslop HE, Moen RC, Ihle JN and Brenner MK (1994) Direct demonstration that autologous bone marrow transplantation for solid tumors can return a multiplicity of tumorigenic cells. *Blood* **84**: 380–383

Schulze R, Schulze M, Wischnik A, Ehnle S, Doukas K, Behr W, Ehret W and Schlimok G (1997) Tumor cell contamination of peripheral blood stem cell transplants and bone marrow in high-risk breast cancer patients. *Bone Marrow Transplant*. **19**: 1223–1228

Spyridonidis A, Schmidt M, Bernhardt W, Papadimitriou A, Azemar M, Wels W, Groner B and Henschler R (1998) Purging of mammary carcinoma cells during ex vivo culture of CD34+ hematopoietic progenitor cells with recombinant immunotoxins. *Blood* **91**: 1820–1827

Vannucchi AM, Bosi A, Glinz S, Pacini P, Linari S, Saccardi R, Alterini R, Rigacci L, Guidi S, Lombardini L, Longo G, Mariani MP and Rossi-Ferrini P (1998) Evaluation of breast tumour cell contamination in the bone marrow and leukapheresis collections by RT-PCR for cytokeratin-19 mRNA. *Br J Haematol* **103**: 610–617

Vogel W, Behringer D, Scheding S, Kanz L and Brugger W (1996) Ex vivo expansion of CD34+ peripheral blood progenitor cells: implications for the expansion of contaminating epithelial tumor cells. *Blood* **88**: 2707–2713

Vredenburgh JJ, Silva O, Broadwater G, Berry D, DeSombre K, Tyer C, Petros WP, Peters WP and Bast RC, Jr. (1997) The significance of tumor contamination in the bone marrow from high-risk primary breast cancer patients treated with high-dose chemotherapy and hematopoietic support. *Biol Blood Marrow Transplant* **3**: 91–97

*© 2001 Cancer Research Campaign*
